# Radiosensitivity of colorectal cancer to ^90^Y and the radiobiological implications for radioembolisation therapy

**DOI:** 10.1088/1361-6560/ab23c4

**Published:** 2019-07-05

**Authors:** Boon Q Lee, Elliot M Abbott, Sarah Able, James M Thompson, Mark A Hill, Christiana Kartsonaki, Katherine A Vallis, Nadia Falzone

**Affiliations:** 1 CRUK/MRC Oxford Institute for Radiation Oncology, Department of Oncology, University of Oxford, Oxford, United Kingdom; 2 Nuffield Department of Population Health, Oxford University, Oxford, United Kingdom; 3 Joint first authors.; 4 Joint last authors.; 5 Author to whom any correspondence should be addressed.; nadia.falzone@oncology.ox.ac.uk; boon.lee@oncology.ox.ac.uk

**Keywords:** colorectal cancer, ^90^Y SIRT, radioembolisation, radiobiology, dosimetry

## Abstract

Approximately 50% of all colorectal cancer (CRC) patients will develop metastasis to the liver. ^90^Y selective internal radiation therapy (SIRT) is an established treatment for metastatic CRC. There is still a fundamental lack of understanding regarding the radiobiology underlying the dose response. This study was designed to determine the radiosensitivity of two CRC cell lines (DLD-1 and HT-29) to ^90^Y *β*^−^ radiation exposure, and thus the relative effectiveness of ^90^Y SIRT in relation to external beam radiotherapy (EBRT).

A ^90^Y-source dish was sandwiched between culture dishes to irradiate DLD-1 or HT-29 cells for a period of 6 d. Cell survival was determined by clonogenic assay. Dose absorbed per ^90^Y disintegration was calculated using the PENELOPE Monte Carlo code. PENELOPE simulations were benchmarked against relative dose measurements using EBT3 GAFchromic^™^ film. Statistical regression based on the linear-quadratic model was used to determine the radiosensitivity parameters 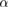 and 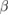 using *R*. These results were compared to radiosensitivity parameters determined for 6 MV clinical x-rays and ^137^Cs *γ*-ray exposure. Equivalent dose of EBRT in 2 Gy (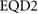) and 10 Gy (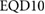) fractions were derived for ^90^Y dose.

HT-29 cells were more radioresistant than DLD-1 for all treatment modalities. Radiosensitivity parameters determined for 6 MV x-rays and ^137^Cs *γ*-ray were equivalent for both cell lines. The 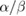 ratio for ^90^Y *β*^−^-particle exposure was over an order of magnitude higher than the other two modalities due to protraction of dose delivery. Consequently, an ^90^Y SIRT absorbed dose of 60 Gy equates to an 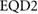 of 28.7 and 54.5 Gy and an 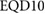 of 17.6 and 19.3 Gy for DLD-1 and HT-29 cell lines, respectively.

We derived radiosensitivity parameters for two CRC cell lines exposed to ^90^Y *β*^−^-particles, 6 MV x-rays, and ^137^Cs *γ*-ray irradiation. These radiobiological parameters are critical to understanding the dose response of CRC lesions and ultimately informs the efficacy of ^90^Y SIRT relative to other radiation therapy modalities.

## Introduction

The success of external beam radiotherapy (EBRT) can partly be attributed to a fundamental understanding of the underlying radiobiology and how this explains the dose response. The evolution of targeted radionuclide therapy (TRT), however, is marked by a recognised deficiency in dose quantification and sound radiobiological understanding. In addition, dosimetry and treatment planning are mostly standardised for EBRT, which is not the situation for TRT (Lassmann *et al* 2011, Gill *et al* 2017). In the case of ^90^Y-based selective internal radiation therapy (^90^Y SIRT), a liver-directed treatment for palliative control of inoperable or chemorefractory tumours (van den Hoven *et al* 2016), doses are usually prescribed using tables or the Body Surface Area method to determine the amount of activity (MBq) to administer (Vauthey *et al* 2002). Absorbed doses reported in literature can vary from 50 up to 200 Gy (Strigari *et al* 2010, Cremonesi *et al* 2014, van den Hoven *et al* 2016). Consequently, there is a general acknowledgement that patient-specific dosimetry needs to be performed to optimise treatment, especially since a dose effect has been established for ^90^Y SIRT (Strigari *et al* 2010, Cremonesi *et al* 2014, van den Hoven *et al* 2016). Given the recent multicentre phase III trial showing that ^90^Y SIRT provides better tumour control within the liver when used in conjunction with chemotherapy than chemotherapy alone (Wasan *et al* 2017), this treatment option could be extended if dosimetric and radiobiological considerations are taken into account in treatment planning.

While there has been a concerted effort to integrate dosimetry into the clinic (Giammarile *et al* 2011), this has not been extended to the incorporation of radiobiological parameters specific for ^90^Y SIRT. Radiobiological modelling based on the linear-quadratic model (LQM) requires detailed knowledge of ^90^Y-specific radiobiological parameters ($\alpha $ and $\beta $) (Cremonesi *et al* 2008). Yet, radiobiological parameters used for ^90^Y SIRT dosimetry planning are usually taken from EBRT studies (Chiesa *et al* 2015). These extrapolated parameters might not be representative of ^90^Y SIRT as they do not account for the intrinsic cellular response to ^90^Y *β*^−^-particles. Furthermore, the protraction of dose delivery for ^90^Y SIRT adds another level of complexity. This effect is encapsulated by the Lea–Catcheside model of sublethal-damage repair (Dale 2018) and has previously been used to describe the *in vitro* cellular response to protracted photon exposure (Solanki *et al* 2017). However, studies using ^90^Y are needed to answer fundamental questions regarding differences in the radiobiological response to ^90^Y *β*^−^-particles and photons of clinically relevant energy.

Recently, Gholami and colleagues (Gholami *et al* 2018) compared cell-kill responses between ^90^Y and EBRT. Using a colorimetric cell viability assay (MTS), they concluded that ^90^Y is less potent than EBRT, as  ≈56 Gy ^90^Y dose cumulated after 8 d was found to be radiobiologically equivalent to a single fraction of  ≈8 Gy EBRT. It is plausible to consider the cumulated dose after ^90^Y exposure as a large number of infinitesimally small doses delivered per fraction. It is thus possible to relate the biological effect of ^90^Y and EBRT by using the radiobiological measure, biological effective dose (BED), which allows inter-comparison between different fractionation schedules or treatment modalities to achieve a given biological effect (Dale *et al* 1996). This concept is only valid though for tissue characterised by a specific $\alpha /\beta $ ratio. Thus, to enable the incorporation of radiobiological parameters such as BED into clinical dosimetry planning of ^90^Y SIRT, accurate measurements of the $\alpha $ and $\beta $ parameters are required to establish equivalence to that of EBRT.

In the present study, we determined the radiobiological parameters, $\alpha $ and $\beta $ for colorectal cancer (CRC) cell lines by means of the clonogenic assay. CRC cells were exposed to three radiation sources, namely ^90^Y *β*^−^-particles (933 keV mean energy and LET range of 0.07–2 keV *μ*m^−1^), 6 MV x-rays (LET  =  0.2 keV *μ*m^−1^) delivered via a clinical linear accelerator (LINAC), and ^137^Cs (662 keV *γ*-ray, LET  =  0.8 keV *μ*m^−1^). Additionally, we investigated the relationship between EBRT and ^90^Y dose through the concept of BED and establishing equivalent EBRT dose of 2 Gy (${\rm EQD2}$) and 10 Gy (${\rm EQD10}$) fractions.

## Materials and methods

### Cell culture

Two CRC cell lines, namely DLD-1 and HT-29, were obtained from American Type Culture Collection. The cells were cultured in Dulbecco’s modified Eagle’s medium (Gibco, Thermo Fisher Scientific, UK), supplemented with 10% foetal calf serum (Merck, UK), and penicillin/ streptomycin/glutamine solution at 100 units ml^−1^, 100 *μ*g ml^−1^ and 0.29 mg ml^−1^, respectively (Gibco, Thermo Fisher Scientific, UK) and were incubated at 37 °C in 5% CO_2_.

### ^90^Y formulations

In this study, ^90^Y either as ^90^Y-DOTATATE or ^90^YCl_3_ was placed in a separate dish to irradiate cells via the long range *β*^−^ emissions of ^90^Y. Since there was no cellular internalisation of ^90^Y, the use of two ^90^Y formulations was not expected to yield different radiobiological effects. ^90^Y-DOTATATE was provided by the radiopharmacy at the Churchill Hospital, Oxford, at 0.05 MBq *μ*l^−1^. ^90^YCl_3_ was purchased from Perkin Elmer (Massachusetts, USA) at 9–10 MBq *μ*l^−1^.

### Clonogenic assays

#### Colony formation and counting

For all experiments, irradiated cells formed colonies in 6-well plates for 7 d (DLD-1) and 9 d (HT-29). Colonies (⩾50 cells) were then fixed and stained with methylene blue in 50% ethanol and counted using an automated colony counter (GelCount^™^, Oxford Optronix Ltd, UK). All experiments were repeated in triplicate.

#### ^90^Y *β*^−^-particles

**Figure 1.**
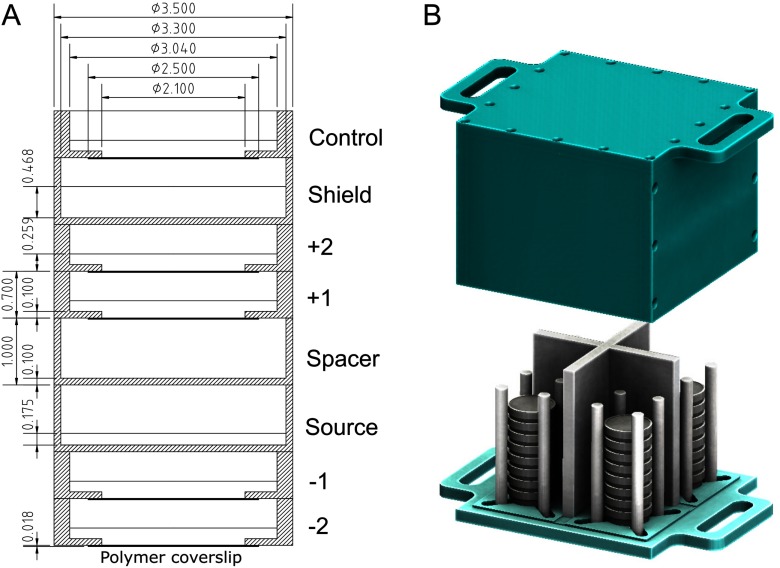
(A) A schematic of a stack of culture dishes used for irradiating plated cells (cell treatment dishes labelled as ‘  +1’, ‘  +2’, ‘  −1’, ‘  −2’ and ‘control’) with ^90^Y (‘source’ dish). The first dish above the source dish was an empty dish (‘spacer’) used to achieve the desired dose in  +1 and  +2 treatment dishes. PBS (1.5 ml) or culture medium (1.5 ml) was added to the source dish and cell-containing dishes, respectively. The ‘shield’ dish was filled with 4 ml of PBS to shield the control dish. A Greiner^®^ 35 mm dish was used for the source, spacer, and shield dishes while an ibidi^®^ low 35 mm *μ*-dish was used for cell dishes. All dimensions shown are in cm. (B) Up to four stacks are positioned inside a custom-made experimental container, before being placed inside an incubator. See supplemental figure 1 for a detailed description of the container.

Stacks of dishes were constructed as shown in figure 1(A). This geometry, adapted from Howell *et al* (1991), allowed the simultaneous irradiation of multiple dishes resulting in different cumulative doses at different dishes within a stack. In addition, it circumvented the problem of cellular internalisation of ^90^Y. Cells were plated at 4000–20 000 cells/dish in 1.5 ml medium on the polymer coverslip of ibidi^®^ low 35 mm *μ*-dishes so that the cells remained within the central area (diameter of 21 mm) while ^90^Y sources were mixed with phosphate buffered saline (PBS) in 1.5 ml of solution in a Greiner^®^ 35 mm dish. The use of different dish types for cells and for ^90^Y was to minimise the difference between doses at the centre and the edge of dish thus assuring a near-uniform dose distribution over the irradiated cells (see supplemental figure 2 (stacks.iop.org/PMB/64/135018/mmedia)). Cell stacks were placed inside a custom-made container (figure 1(B)) and exposed for 6 d inside an incubator with 0, 10, 15, and 20 MBq of ^90^Y diluted from stock. After the exposure period, the cells were replated at 3000–5000 cells/well in 1.5 ml of medium in three wells on 6-well plates. The cumulated doses delivered ranged from 0–32 Gy. Radiation dose was delivered at variable average dose rates ranging from 0–0.0037 Gy min^−1^.

#### ^137^Cs *γ*-ray

Cells were plated at 1000–40 000 cells/well in three wells on 6-well plates and irradiated 4 h after plating with a caesium irradiator (IBL637, CIS Bio international, France) at doses ranging from 0–10 Gy. Radiation dose was delivered at 0.77 Gy min^−1^.

#### LINAC 6 MV x-rays

The cells were plated at 1000–10 000 cells/well in three wells on 6-well plates 4 h prior to the treatment delivery. A Varian Clinac 2100 series was used to deliver 0–10 Gy at 6.6 Gy min^−1^ using a 15  ×  15 cm^2^ field size. The gantry was positioned at 180° such that the beam first passed bottom-up through the couch, followed by 1.5 cm of solid water so that the dose maximum was at the cell level, and finally 2 cm of solid water to capture the backscatter dose.

### Monte Carlo (MC) modelling

The dose absorbed per ^90^Y disintegration, $S$ (cGy MBq^−1^ d^−1^), in a cell monolayer 15 *μ*m in height (water density, *ρ*  =  1.0 g cm^−3^ was assumed) contiguous with the bottom of each ibidi^®^ dish placed at different positions above or below the source dish (figure 1(A)) was calculated by the MC method using the PENELOPE code (Salvat *et al* 2011). The average cell height was measured using confocal microscopy (supplemental table 1). Polystyrene (*ρ*  =  1.06 g cm^−3^) was assumed for both Greiner^®^ and ibidi^®^ dishes and air of 95% humidity at 37 °C and 5% CO_2_ (*ρ*  =  1.276  ×  10^−3^ g cm^−3^) was used for air inside each dish. PBS and ^90^Y solution were assumed as water. The *β*^−^ spectrum of ^90^Y was taken from medical internal radiation dose (MIRD) tabulation (Eckerman and Endo 2008). A total of 10^8^ primaries were simulated in each run. All primaries and secondaries were followed until their energies reached  <1 keV and their remaining energies were assumed to be deposited locally.

Up to a third of the initial medium volume in each dish was lost by the end of the exposure period due to evaporation. For the source dish and all treatment dishes, two *S*-values were calculated based on the measured medium volume at the beginning (${{S}_{initial}}$) and conclusion (${{S}_{final}}$) of the exposure period. *S*-values in between ${{S}_{initial}}$ and ${{S}_{final}}$ were interpolated linearly. As a result, the MIRD formulation (Goddu *et al* 1997) was modified to account for the evaporation effect in each dish and the final equation used for dose calculation is (see supplemental material for derivation, equations (S1)–(S3)):

1 \begin{align*} \newcommand{\e}{{\rm e}} \displaystyle D\left( T \right)=\frac{{{A}_{0}}}{\lambda }\left[ \frac{{{S}_{final}}-{{S}_{initial}}}{\lambda T}\left( 1-\left( 1+\lambda T \right){{e}^{-\lambda T}} \right)+{{S}_{initial}}\left( 1-{{e}^{-\lambda T}} \right) \right]\nonumber \end{align*}

where $D\left( T \right)$ is the dose absorbed for cells exposed to ^90^Y for duration $T$ with an initial activity of ${{A}_{0}}$ and $\lambda $ is the physical decay constant of ^90^Y ($\lambda ={\rm ln}2/{{T}_{phys}}$ and physical half-life ${{T}_{phys}}=64.1$ h).

### ^90^Y dose calibration

**Figure 2.**
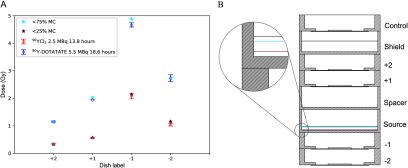
(A) Comparison of the measured dose distribution in ‘dry’ conditions determined using EBT3 film with MC simulations. Error bars represent the standard deviations calculated from the two experiments for each type of radiopharmaceutical. Measured dose distributions for ^90^YCl_3_ (in red) and ^90^Y-DOTATATE (in blue) closely agreed with MC simulated dose distributions when non-uniform ^90^Y source distributions were assumed. Two simulated dose distributions are shown. The first assumes exposure to 5.5 MBq of ^90^Y for 18.6 h exposure with a concentration gradient in the bottom 75% of the source solution (in cyan, labelled as ‘<  75% MC’). The second assumes exposure to 2.5 MBq of ^90^Y for 13.8 h with a concentration gradient in the bottom 25% of the source solution (in purple, labelled as ‘<  25% MC’). (B) Schematic diagram of the stack geometry used for calibration. In the expanded circular view, two coloured horizontal lines represent the aforementioned ^90^Y concentration gradients used in the simulations.

A series of calibration experiments was performed to quantify the ^90^Y distribution. Radiochromic films (8.2  ×  9.7 mm; GAFchromic^™^ EBT3 film: Ashland Inc., Covington, KY) were exposed to 6 MV x-rays to doses ranging from 0.5–10 Gy in accordance with the AAPM TG-61 protocol (Ma *et al* 2001). For ^90^Y exposure, the EBT3 films were placed centrally in the treatment dishes (i.e. numbered dishes in figure 1(A)), and exposed for 14–19 h. Exposures were done with the films *in situ* in dry conditions, due to the solubility of the films, and not submerged in medium to replicate the conditions of cell exposure. Experiments were performed in duplicate for both ^90^Y-DOTATATE and ^90^YCl_3_. EBT3 films exposed to 6 MV x-rays and ^90^Y were scanned 24 h after irradiation, using an Epson Expression 10000 XL colour scanner in transmission mode. A calibration curve relating the dose reading to x-ray dose was derived from the 6 MV LINAC data and this was used to inform the dose achieved from the ^90^Y exposure (Technical-Report 2010). A MC simulation (figure 2(B)) emulating the geometry previously described (figure 1(A)) was used to calculate the dose to the EBT3 film. Absolute dose measurements were determined by benchmarking the EBT3 film determined doses across all treatment dishes against the PENELOPE simulation (figure 2(A)). For the MC simulation, the material composition and density of the EBT3 film was based on previously reported values (Fiorini *et al* 2014). The absolute doses were background-subtracted based on the measured dose for the control dish.

### Radiobiological modelling

We can relate different fractionation schemes in terms of the LQM by using the BED concept (Dale 1996). For a fractionated EBRT treatment of $d$ Gy per fraction (not accounting for repopulation), BED is given by:

2 \begin{align*} \newcommand{\e}{{\rm e}} \displaystyle {\rm BE}{{{\rm D}}_{{{\left( \alpha /\beta \right)}_{EBRT}}}}=nd\left( 1+\frac{d}{{{\left( \alpha /\beta \right)}_{EBRT}}} \right)\nonumber \end{align*}

where $n$ is the number of fractions and ${{(\alpha /\beta)}_{EBRT}}$ is an inverse measure of tissue sensitivity to changes in fractionation or dose rate. However, for the protracted dose delivery of ^90^Y SIRT, sublethal damage repair can take place during the irradiation period, making the treatment less effective. Furthermore, radiation of different linear energy transfer (LET) may yield different relative biological effectiveness (RBE). A modified BED equation that accounts for LET and dose-rate effects can be used for ^90^Y radiation (Dale and Jones 1999):

3 \begin{align*} \newcommand{\e}{{\rm e}} \displaystyle {\rm BE}{{{\rm D}}_{^{90}{\rm Y},{{\left( \alpha /\beta \right)}_{EBRT}}}}={{D}_{^{90}{\rm Y}}}\left( {\rm RB}{{{\rm E}}_{max}}+\frac{{{G}_{\infty }}{{D}_{^{90}{\rm Y}}}}{{{\left( \alpha /\beta \right)}_{EBRT}}} \right)\nonumber \end{align*}

where ${{D}_{^{90}{\rm Y}}}$ is the cumulative dose of ^90^Y radiation, ${\rm RB}{{{\rm E}}_{max}}={{\alpha }_{^{90}{\rm Y}}}/{{\alpha }_{EBRT}}$ is the maximum (or intrinsic) RBE at zero dose, and $G$ is the Lea–Catcheside dose-protraction factor. $G$ can be estimated for a fully-decayed radiation source as:

4 \begin{align*} \newcommand{\e}{{\rm e}} \displaystyle {{G}_{\infty }}=\frac{{{T}_{rep}}}{{{T}_{rep}}+{{T}_{phys}}}\nonumber \end{align*}

where ${{T}_{rep}}$ and ${{T}_{phys}}$ are the sublethal damage repair half-time and radionuclide decay half-life, respectively. For a finite exposure, $G$ can be determined from supplemental equation (S4). This modified BED formalism assumes that the intrinsic quadratic component ($\beta $) remains unchanged between modalities and the dose-rate effect is encapsulated by the $G$ factor resulting in a lower magnitude of the second term of equation (3). To compare relative efficacy of different fractionation schedules and different radiation modalities, it is useful to invoke the concept of equivalent dose in fractionated EBRT (EQD) (Fowler 2009). EQD for ^90^Y physical dose, ${{D}_{^{90}{\rm Y}}}$, absorbed during a SIRT treatment is (see supplemental equation (S5) for derivation):

5 \begin{align*} \newcommand{\e}{{\rm e}} \displaystyle {\rm EQ}{{{\rm D}}_{{{\left( \alpha /\beta \right)}_{EBRT}}}}=\frac{{{D}_{^{90}{\rm Y}}}\cdot \left( {\rm RB}{{{\rm E}}_{max}}+\frac{{{G}_{\infty }}{{D}_{^{90}{\rm Y}}}}{{{\left( \alpha /\beta \right)}_{EBRT}}} \right)}{1+\frac{d}{{{\left( \alpha /\beta \right)}_{EBRT}}}}.\nonumber \end{align*}

### Statistics

The $\alpha /\beta $ values were estimated by fitting a linear mixed-effects model by restricted maximum likelihood. Parameters $\alpha $ and $\beta $ were estimated from the surviving fraction (SF), $-{\rm ln}\left( {\rm SF} \right)\,=$ $\alpha D+\beta {{D}^{2}}$. A random intercept was included for sets of replicates to consider the dependence between replicates. The model was fitted to data for each cell line separately. Approximate standard errors of $\alpha /\beta $ were calculated as $\sqrt{\left( \left( 1/{{\beta }^{2}} \right){\rm var}\left( \alpha \right)-( {2\alpha }/{{{\beta }^{3}}})\, {\rm cov}\left( \alpha ,~\beta \right)+ \left( {{\alpha }^{2}}/{{\beta }^{4}} \right){\rm var}\left( \beta \right) \right)}$. Wald tests were used to assess whether the estimated $\alpha $ and $\beta $ were significantly different from zero. The R software package (version 3.3.3, R Core Team (2017)) and package nlme (Pinheiro *et al* 2017) were used for statistical analysis.

## Results

### Dosimetry

PENELOPE simulations were performed for four hypothetical ^90^Y concentration gradients where ^90^Y was assumed to be uniformly distributed in the bottom 25%, 50%, 75%, and 100% of the source solution. EBT3 film measured and MC simulated relative doses were compared (supplemental figure 3). Results show that the dishes were asymmetrically affected by the dose gradient, based on whether the dishes were above or below the source. Furthermore, the  −1 and  −2 dishes were much more sensitive to the dose gradient, whereas relative doses determined from MC simulation and EBT3 film measurements were consistent for the  +1 and  +2 dishes (i.e. all dose points are superimposed on the graph). The comparison suggests that the ^90^Y activity was concentrated at the bottom 75% and 25% of the source solution for ^90^Y-DOTATATE and ^90^YCl_3_, respectively. Figure 2 shows that there was good agreement (<10% difference) between the measured absolute dose from calibration experiments compared with MC simulated results when these non-uniform ^90^Y source distributions were assumed. The non-uniformity of the ^90^Y source may be attributed to the chemical interactions between the free ^90^Y^3+^ ions and the PBS used to dilute the ^90^Y activity. This is supported by the fact that free ^90^Y in ^90^YCl_3_ exhibited more extreme non-uniformity in distribution than chelated ^90^Y in ^90^Y-DOTATATE and that precipitation was observed visible when cold YCl_3_ and PBS were mixed at high concentration. These experimentally determined source distributions were included in the subsequent dose calculations for clonogenic experiments. Table 1 compares the cell monolayer ${{S}_{initial}}$-values calculated for ^90^YCl_3_ and ^90^Y-DOTATATE assuming a gradient distribution of ^90^Y in the bottom 25% and 75% of the source dishes, respectively.

**Table 1.** Comparison of MC calculated ${{S}_{initial}}$-values for the 15 *μ*m-thick cell monolayer in each treatment dish exposed to either ^90^YCl_3_ or ^90^Y-DOTATATE.

Dish position	${{S}_{initial}}$-value (cGy MBq^−1^ d^−1^)	
	^90^YCl_3_	^90^Y-DOTATATE
+2	6.26	8.40
+1	37.9	47.3
−1	31.4	24.3
−2	3.01	2.06

### Clonogenic assays

**Figure 3.**
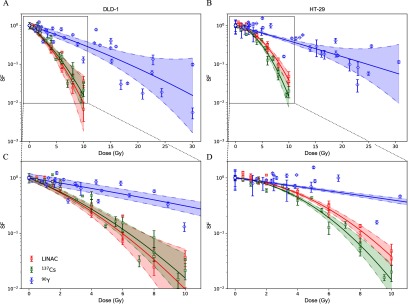
Experimental surviving fractions of (A) DLD-1 and (B) HT-29 cells irradiated by LINAC (red), ^137^Cs (green), and ^90^Y (blue). Surviving fraction based on the $\alpha $ and $\beta $ values estimated from fitting a linear mixed-effects model by restricted maximum likelihood are shown (solid lines). The shaded area represents the 95% confidence interval (CI) of the fit. Each data point with its associated error bar is the mean  ±  standard deviation derived from three biological repeats in a single replicate. Figures (C) and (D) show the close-up in the first 10 Gy.

Figure 3 compares the experimental surviving fractions for DLD-1 and HT-29 cells exposed to ^90^Y *β*^−^-particles, clinical 6 MV x-rays, and ^137^Cs *γ*-ray. The HT-29 cell line was more radioresistant towards all radiation sources compared with DLD-1 within the dose range considered and this was consistent with previously published results for ^137^Cs *γ*-ray irradiation (Gao *et al* 2009). 6 MV x-rays and ^137^Cs *γ*-ray induced an almost identical radiobiological response from both cell lines but HT-29 was slightly more sensitive to ^137^Cs *γ*-ray at high doses. Table 2 summarises the fitted $\alpha $, $\beta $ and $\alpha /\beta $ values for the survival curves. For ^90^Y *β*^−^-particles, the measured $\beta $ parameter encapsulates repair that took place during the 6 d exposure period. The $\alpha $ and $\beta $ parameters for EBRT derived from our methods were consistent with previously reported values (Miura *et al* 2012, Gholami *et al* 2018). It is noteworthy that the estimated $\alpha $ parameter for HT-29 exposed to ^90^Y *β*^−^-particles was slightly higher than other modalities, though the 95% confidence intervals (CIs) for the estimated $\alpha $ for 6 MV x-rays and ^137^Cs *γ*-ray are overlapped significantly with the 95% CI of the estimated $\alpha $ for ^90^Y *β*^−^-particles. In contrast, DLD-1 exposed to ^90^Y *β*^−^-particles exhibited less than half of the linear-term radiosensitivity towards 6 MV x-rays and ^137^Cs *γ*-ray. $\beta $ values for ^90^Y were very small due to the protracted dose delivery and this led to considerably larger $\alpha /\beta $ ratios.

**Table 2.** Comparison of $\alpha $, $\beta $, and $\alpha /\beta $-values derived from LQM fitting of the survival curves for DLD-1 and HT-29 cells exposed to either 6 MV x-rays (LINAC), ^137^Cs *γ*-ray or ^90^Y *β*^−^-particles. The range shown within brackets represents the 95% CIs of the estimated parameter.

Cell line	Radiation source	*α* (Gy^−1^)	$\beta $ (Gy^−2^)	$\alpha /\beta $ (Gy)
DLD-1	LINAC	0.273	0.0189	14.4
		(0.187–0.359)	(0.009 70–0.0282)	(3.15–25.7)
	^137^Cs	0.264	0.0153	17.3
		(0.198–0.330)	(0.008 33–0.0222)	(5.37–29.1)
	^90^Y	0.106	0.00109	97.0
		(0.075–0.137)	(−0.000 122–0.00230)	(36.8–231)
	^90^Y^a^	0.129	0	N/A
		(0.114–0.144)		
HT-29	LINAC	0.050	0.0276	1.81
		(0.008–0.092)	(0.0230–0.0323)	(0.0247–3.60)
	^137^Cs	0.056	0.0367	1.54
		(0.003 43–0.109)	(0.0304–0.0429)	(−0.122–3.19)
	^90^Y	0.090	0.000141	637
		(0.063–0.116)	(−0.000 969–0.00125)	(−4517–5792)
	^90^Y^a^	0.0897	0	N/A
		(0.0792–0.100)		

a Additional fit using only the linear $\alpha $ component since $\beta $ from the standard LQM is consistent with zero.

Radiobiological parameters, ${\rm RB}{{{\rm E}}_{max}}$ and $G$, used in the derivation of equation (3) for $\alpha /\beta $-ratios provided in table 2, are summarised in table 3. If we adopt the premise that the *β* parameter for ^90^Y is equal to that of EBRT by incorporating the dose protraction factor *G*, we can determine ${{G}_{\infty }}$ for a fully-decayed source by solving ${{G}_{T}}={{\beta }_{^{90}{\rm Y}}}/{{\beta }_{EBRT}}$ for ${{T}_{rep}}$ (see ${{G}_{T}}$ in equation S4 in supplemental material) and applying the derived ${{T}_{rep}}$ to equation (4).

**Table 3.** Comparison of ^90^Y SIRT radiobiological modelling parameters derived for each cell line from LQM fitted parameters in table 2. The repair half-time ${{T}_{rep}}$ was determined using supplemental equation (S4). ${{G}_{\infty }}$ was estimated based on the derived ${{T}_{rep}}$ and equation (4). The last column shows the ${{\left( \alpha /\beta \right)}_{^{90}{\rm Y}}}$ for indefinite exposure. The range shown within brackets represents the 95% CI of the estimated parameter.

Cell line	${\rm RB}{{{\rm E}}_{max}}$ (${{\alpha }_{^{90}{\rm Y}}}/~{{\alpha }_{LINAC}}$)	${{G}_{6}}$ (${{\beta }_{^{90}{\rm Y}}}/{{\beta }_{LINAC}}$)	${{T}_{rep}}$ (h)	${{G}_{\infty }}$	${{\alpha }_{^{{\rm 90}}{\rm Y}}}/{{G}_{\infty }}{{\beta }_{LINAC}}~$ (Gy)
DLD-1	0.388	0.0577	2.51	0.0377	148
	(0.221–0.555)	(−0.0123–0.1277)	(−0.67–5.69)	(−0.0102–0.0856)	(−56.3–353)
HT-29	1.800	0.0051	0.21	0.0033	979
	(0.198–3.402)	(−0.0351–0.0453)	(−1.48–1.90)	(−0.0230–0.0296)	(−6911–8861)

### Radiobiological modelling

**Figure 4.**
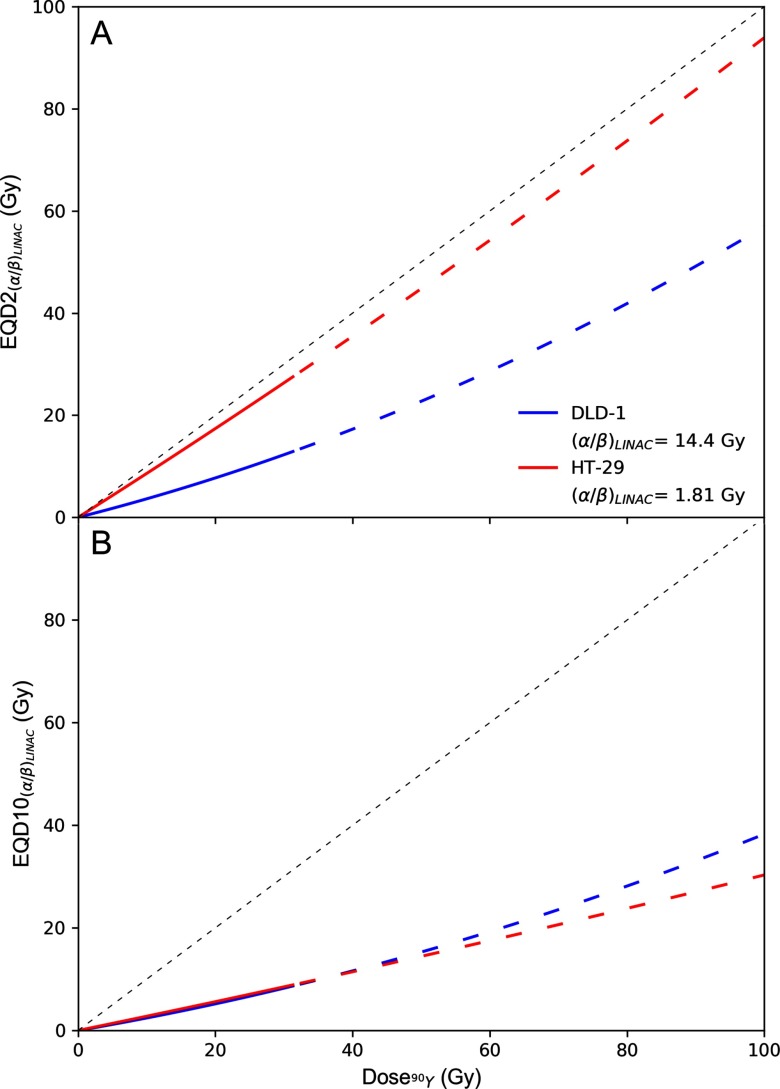
(A) Equivalent EBRT dose in 2 Gy fractions, ${\rm EQD}{{{\rm 2}}_{{{\left( \alpha /\beta \right)}_{EBRT}}}}$, as a function of ^90^Y absorbed dose (unfractionated and fully-decayed) for DLD-1 (blue) and HT-29 (red) cell lines. (B) Equivalent EBRT dose in 10 Gy fractions, ${\rm EQD1}{{{\rm 0}}_{{{\left( \alpha /\beta \right)}_{EBRT}}}}$. For ^90^Y dose greater than 32 Gy, EQD is plotted as a dashed line to indicate that these values were extrapolated from measurements acquired at lower doses (<32 Gy).

Figure 4 shows ${\rm EQD}{{{\rm 2}}_{\alpha /{{\beta }_{LINAC}}}}_{~}$(top panel) and ${\rm EQD1}{{0}_{\alpha /{{\beta }_{LINAC}}}}_{~}$ (bottom panel) of ^90^Y physical dose calculated using equation (5) and the radiosensitivity parameters presented in table 2. Physical dose of ^90^Y was extrapolated to 100 Gy assuming an increased initial dose rate is not expected to significantly modify the intrinsic radiosensitivity of DLD-1 and HT-29 towards ^90^Y *β*^−^ radiation. This assumption is supported by a previous study of DLD-1 and HT-29 that showed the $\alpha $ terms were almost equivalent following exposure to 0.25 and 42 Gy h^−1^ of ^137^Cs irradiation (Williams *et al* 2008). The figure shows ^90^Y SIRT would be less effective than EBRT delivered in 2 Gy fractions for treating the DLD-1 cell line because it exhibits a high $\alpha /\beta $ ratio when exposed to LINAC *x*- or ^137^Cs *γ*-radiation and a low $\alpha $ value when exposed to ^90^Y *β*^−^ radiation. Extrapolating from the fit parameters, it requires  ≈100 Gy of ^90^Y dose to achieve the same biological cell-killing effect as an EBRT of 30 fractions of 2 Gy. In contrast, HT-29 would respond to each Gy of ^90^Y similarly to each Gy of EBRT delivered in 2 Gy fractions. However, EBRT delivered in 10 Gy fractions was more potent in treating either cell line than ^90^Y SIRT. It is noteworthy that the accuracy of the predicted EQD is affected by the uncertainties of the estimated radiobiologic parameters (supplemental figure 4).

## Discussion

With the increased use of TRT such as ^90^Y SIRT for liver cancer, there is a pressing need to incorporate radiobiologic information into the decision-making process to optimise dose given during multiple administrations or to combine ^90^Y SIRT with EBRT, specifically, stereotactic ablative radiotherapy (SABR) for curative intent. Here, the survival of DLD-1 and HT-29 CRC cells after exposure to ^90^Y *β*^−^-particles, 6 MV x-rays, and ^137^Cs *γ*-ray were determined by clonogenic assay. Radiosensitivity parameters, $\alpha $ and $\beta ,$ derived from the fitted survival curves were then used to calculate the equivalent dose in fractionated EBRT for ^90^Y SIRT.

The experimental setup shown in figure 1 was designed to simulate the mechanism of dose delivery by ^90^Y SIRT, where ^90^Y-loaded microspheres are permanently trapped at the arteriolar end of the capillary bed and not internalised into cancer cells. This setup physically isolated the ^90^Y from the cells, which contrasts with the recent investigation by Gholami *et al* (2018), where cells were mixed with ^90^YCl_3_ in a 96-well plate. Using the MTS assay, they determined metabolic viability curves for three CRC cell lines (HT-29, HCT-116 and SW-48), exposed to ^90^Y *β*^−^-particles and 6 MV x-rays. The respective $\alpha $ and $\beta $ parameters of HT-29 derived from the metabolic viability curves were 0.0842 Gy^−1^ and 0.0239 Gy^−2^ for EBRT and 0.0145 Gy^−1^ and 0.0005 Gy^−2^ for ^90^YCl_3_. Although the MTS assay measures metabolic viability rather than reproductive cell death, the $\alpha $ and $\beta $ parameters for EBRT agree within the 95% CI of our estimated values. However, results for ^90^Y diverge. Gholami *et al* found that  ≈56 Gy of ^90^Y dose (cumulated after 8 d) is necessary to decrease metabolic viability to achieve the same cell kill as a single fraction of 8 Gy EBRT. Our results from clonogenic survival suggest a much lower ^90^Y dose (cumulated after 6 d) of 20.7 Gy for DLD-1 and 23.6 Gy for HT-29 is necessary. For indefinite exposure, these values are 22.9 and 23.8 Gy, respectively. This discrepancy could be due to the differences in the two assays or the assumption that ^90^Y did not internalise into the cells and was uniformly distributed throughout the well adopted in Gholami’s study. In contrast, our results show that ^90^Y was not uniformly distributed inside a tissue culture-treated dish and this differential distribution of ^90^Y could affect dose calculations if not corrected.

The protracted dose delivery of ^90^Y significantly increased clonogenic survival compared to acute exposure from either LINAC *x*- or ^137^Cs *γ*-irradiation. Although the dose rate of the LINAC was nine times higher than that of the ^137^Cs irradiator, the radiobiologic responses of DLD-1 and HT-29 towards these modalities were very similar, as both radiation dose deliveries were completed within minutes before damage repair could reduce cell death (Howard *et al* 2017). In contrast to the linear component, the experimental ${{\beta }_{^{{\rm 90}}{\rm Y}}}$ was extremely small for ^90^Y *β*^−^ radiation and was not significantly different from zero by a Wald test (*p*   >  0.05, DLD-1: 0.001 09 (95% CI, −0.000 122–0.002 30) and HT-29: 0.000 141 (95% CI, −0.000 969–0.001 25)), i.e. not contributing statistically significantly to the fitting of the data within the LQM. The highly suppressed ${{\beta }_{^{{\rm 90}}{\rm Y}}}$ was consistent with the predicted $G$ factor for 6 d exposure (<0.06 for an assumed ${{T}_{rep}}$ of 0–5 h) (Dale 1996). The very small quadratic term of the LQM for a low-dose-rate radiation treatment is a well known issue and a simplified version of BED where $\beta =0$, which neglects dose-rate effects, has been used in the literature (Chiesa *et al* 2015). As expected, this $\beta =0$ assumption does not appreciably alter the value of $\alpha $ since the CI for ${{\beta }_{^{90}{\rm Y}}}$ in each cell line contains zero (table 2).

Furthermore by adopting the assumptions that ${{\alpha }_{^{90}{\rm Y}}}={\rm RB}{{{\rm E}}_{max }}~\times {{\alpha }_{EBRT}}$ and ${{\beta }_{^{90}{\rm Y}}}=G\times {{\beta }_{EBRT}}$ (Dale and Jones 1999), we are able to perform additional radiobiological modelling (table 3). Using these assumptions, we were able to extract a physiologically meaningful ${{T}_{rep}}$ of 2.5 h for the DLD-1 cell line and an ${\rm RB}{{{\rm E}}_{max}}~$ of 0.4. Interestingly, the HT-29 cell line was seemingly more sensitive to ^90^Y than either LINAC or ^137^Cs, with an ${\rm RB}{{{\rm E}}_{max}}&gt;1$, although the 95% CIs of their $\alpha $-values overlap significantly. Data for HT-29 from Gholami *et al* (2018) suggest an ${\rm RB}{{{\rm E}}_{max}}~$ of 0.172, but this value is outside the lower CI for HT-29 in the current study. For ${{T}_{rep}}$, the 95% CIs contain negative values for both cell lines and the repair half-time ${{T}_{rep}}$ was only 12 min for HT-29, which was not expected as physiological repair half-times are generally longer. A short ${{T}_{rep}}$ could indicate either the experimental uncertainties are so large that it is not possible to extract a suppressed $\beta $ parameter with high accuracy and thus a physiologically meaningful repair time or the assumption that ‘the *β*-values are equivalent by incorporating the dose protraction factor $G$’ is not valid here. If the latter is correct then, for the HT-29 cell line, we cannot assume that there is a relationship between the radiobiological parameters of ^90^Y and EBRT. It is worthwhile to point out that the latter assumes not only that the $\alpha $ values are related through RBE_*max*_ but that the $\beta $ values are independent of LET. There are other RBE models that predicted a dependence of the $\beta $ value on LET (Stewart *et al* 2018). However, these dependencies were seen mainly in proton and heavier ions at LET values exceeding those of the currently used radiation modalities, that is for LET  >4 keV *μ*m^−1^.

These assumptions need to be tested in other CRC cell lines to determine whether the radiobiology of these cell lines are indeed distinct from that of EBRT. Survival parameters from Gholami *et al* (2018) suggest ${{T}_{rep}}$ values of 1.04, 1.08, and 1.06 h for HCT-116, SW-48, and HT-29 cell lines, respectively. Although acquired through different methods, these values are consistent with each other and lie within the 95% CI of the parameters extracted from this study. Substituting out ${{G}_{6}}$ with our predicted ${{G}_{\infty }}$, determined from ${{T}_{rep}}$ using equation (4), further supressed the $\beta $s for ^90^Y and results in a very high $\alpha /\beta $ ratio. Unless there is a significant biological effect resulting from the cellular internalisation of ^90^Y, the $\alpha $ and $\beta $ parameters derived in this work would still apply to other targeted radionuclide therapies, such as peptide receptor radionuclide therapy using ^90^Y-DOTATATE.

${\rm EQD2}$ and ${\rm EQD10}$ were derived to quantify the relative effectiveness of ^90^Y SIRT compared to EBRT. In the last decade, SABR has been utilised for treating liver metastases of CRC origin (Comito *et al* 2015). Figure 4 shows that SABR with 10 Gy fractions could be more effective than both standard EBRT delivered in 2 Gy fractions and ^90^Y SIRT in treating such disease. However, ^90^Y SIRT could be an attractive alternative to standard EBRT as it has comparable efficacy while sparing healthy tissue due to protraction in dose delivery.

## Conclusions

In this manuscript, we report a comprehensive study in which the radiosensitivity parameters of two CRC cell lines, DLD-1 and HT-29, to ^90^Y *β*^−^-particles in comparison to that of EBRT (6 MV x-rays and *γ*-ray from a ^137^Cs irradiator) were explored. Using statistical regression of the clonogenic survival data within the LQM framework, we conclude that the $\alpha $ values of cells exposed to ^90^Y were significantly different from those exposed to either LINAC or ^137^Cs, whereas the *β* values were not significantly different from zero. In addition, we provide a framework that relates the physical dose required for ^90^Y to yield an equivalent EBRT biological response based on the concept of BED. Accounting for these differences in radiosensitivity enables researchers and clinicians to calculate equivalent doses (EQD) in a combined therapy (^90^Y SIRT and EBRT) setting.
